# A nanogold sensor test for tire wear chemicals based on the plasmon ruler approach

**DOI:** 10.1007/s00604-024-06376-3

**Published:** 2024-05-17

**Authors:** François Gagné, Eva Roubeau-Dumont, Chantale André

**Affiliations:** Environment and Climate Change Canada, Aquatic Contaminants Research Division, 105 McGill, Montréal, QC Canada

**Keywords:** Plasmon resonance, Nanogold sensor, Tire wear substances, Aquatic environment, Bioaccumulation

## Abstract

The release of tire wear substances in the environment is raising concerns about potential impacts on aquatic ecosystems. The purpose of this study was to develop a quick and inexpensive screening test for the following tire wear substances: 6-phenylphenyldiamine quinone (6-PPD quinone), hexamethoxymethylmelamine (HMMM), 1–3-diphenylguanidine (1,3-DPG), and melamine. A dual strategy consisting of nanogold (nAu) signal intensity and the plasmonic ruler principle was used based on the spectral shift from the unaggregated free-form nAu from 525 nm to aggregated nAu at higher wavelengths. The shift in resonance corresponded to the relative sizes of the tire wear substances at the surface of nAu: 6-PPD (560 nm), HMMM (590 nm), 1,3-DPG (620 nm), and melamine (660 nm) in a concentration-dependent manner. When present in mixtures, a large indiscriminate band between 550 and 660 nm with a maximum corresponding to the mean intermolecular distance of 0.43 nm from the tested individual substances suggests that all compounds indiscriminately interacted at the surface of nAu. An internal calibration methodology was developed for mixtures and biological extracts from mussels and biofilms and revealed a proportional increase in absorbance at the corresponding resonance line for each test compound. Application of this simple and quick methodology revealed the increased presence of melamine and HMMM compounds in mussels and biofilms collected at urban sites (downstream city, road runoffs), respectively. The data also showed that treated municipal effluent decreased somewhat melamine levels in mussels.

**Graphical Abstract:**

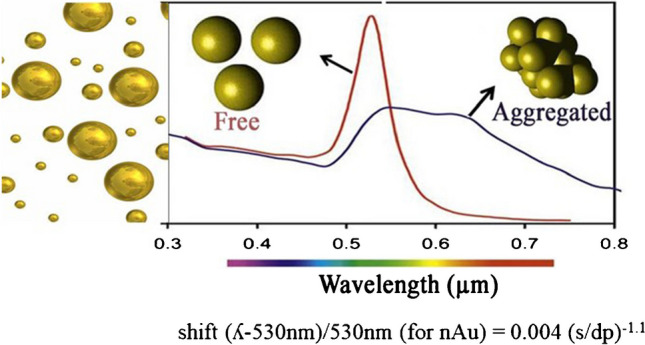

**Supplementary Information:**

The online version contains supplementary material available at 10.1007/s00604-024-06376-3.

## Introduction

The increasing use of tires from vehicle use results in the release of tire wear particles and substances in nearby ecosystems. Indeed, the global car use has increased continuously over the last decade with tire wear emissions reaching between 10^6^ and 10^8^ kg annually worldwide [1]. Tire wear particles are initially found between 70 and 80 µm in size, but dimensions are also found at the nanometer range in simulated environments [2], and become more bioavailable and potentially toxic for organisms. The composition of tire wear substances is complex and contains mainly organic compounds, sulfur, and metals such as zinc and iron. Organic compounds vary in their relative abundance, but the compound 1,3-diphenylguanidine (1,3-DPG) reached 1.8 µg/L in road runoffs and was found at 20 ng/L in surface water [3]. Given the hydrophobic nature of this compound (log octanol-water coefficient-kow of 2.4), the bioaccumulation potential is estimated between 10 and 100, suggesting accumulation on the long term in resident organisms. The compound hexamethoxymethylmelamine (HMMM) can be found at concentrations in the mg/L range similarly as zinc in tire wear leachates [2, 4] and is considered as one of its most abundant compounds, although less toxic based on the low hydrophobicity with a log kow = 1 [5]. HMMM contains a melamine core, which are also found in tire wear albeit at lower concentrations. The compound 6-PPD quinone (6-PPDQ) is also released from tire wear but at much lower concentration than HMMM [6], but with its log kow = 3.9 is much more toxic to fish. Indeed, the lethal concentration of 50% in the range 0.1–1 µg/L was found in salmonids [7]. Given the high amount of tire wear substances released in the environment, these compounds have the potential to harm aquatic and terrestrial ecosystems, especially on the long term. Current methods to measure them in various environmental samples rely on HPLC mass spectrometry, which are expensive and not readily accessible to low-budget laboratories. In this context, the development of quick and inexpensive early warning tests for detection of tire wear substances in organisms and other media is urgently needed.

Gold nanoparticles (nAu) have gained attention for their unique optical properties, making them quick and inexpensive sensing tool. Electromagnetic radiation can interact (resonate) with metal nanoparticles such as nAu from the coherent oscillation of electrons (plasmons) at the surface. While free nAu absorbs at 530 nm (red color), clusters of nAu exhibit a longitudinal surface plasmon resonance at longer wavelengths up to 650–700 nm range (purple color). An attractive feature of nAu sensors is that these spectral shifts (from red to purple) are directly observable by the naked eye and easily analyzed by any smartphone camera capable of measuring red, green, and blue colors using a freely accessible software. On the one hand, the increase in plasmon resonance (absorbance) is related to concentration, while on the other hand, aggregation between nAu shifts the resonance maximum from 530 nm to higher wavelengths as the nanoparticles get closer together during aggregation. Interestingly, the wavelength shift to longer wavelengths is inversely proportional to the interparticle distance (*s*) between two nAu particles at the 1 nm range, i.e., the shift is increased from 530 to > 650 nm as the distance gets smaller between nAu [8]. This relationship is called the plasmon ruler equation and is defined as follows: shift (*ʎ* − 530 nm)/530 nm (for nAu) = 0.004 (*s*/dp)^−1.1^ where *s* corresponds to intermolecular distance between nAu particles and dp the diameter of the nAu and the shift between higher wavelengths *ʎ* and the transversal absorbance of isolated (unaggregated) nAu at 530 nm. Hence, the spectral shift in wavelengths provides information on the relative distance between nAu particles where the intensity remains related to concentration of these assemblages. Aggregation of nAu is either directly promoted by surface charge cancelation by HCl or NaCl. The surface of nAu particles can be modified by various ligands (e.g., arginine for dibutylphthalate, Triton X-100/citrate for melamine, and mercaptoundecanoic acid for polystyrene nanoplastics) enabling the detection of various analytes based on either aggregation or disaggregation [9].

In theory, the relative distance between nAu depends on the composition of surface films of nAu, which can be sensed at the nm scale based on the plasmonic ruler equation with distance ≤ 1 nm. A sensor was developed for melamine compound using Triton X-100/citrate-coated nAu where free nAu (red color, > 650 nm) is aggregated (purple color) in the presence of melamine following the addition of 10 mM HCl [10]. Indeed, when melamine forms a film at the surface of nAu, it reduces the electrostatic repulsion at the surface and favors aggregation by forming bridges with other nAu, which is easily observed by the formation of purple color of the solution. The hypothesis of this study states that the selected tire wear substances mentioned also contain amine groups as with melamine, which should promote aggregation, but at wavelengths related to the size differences of the molecules forming films at the surface of nAu. For example, a larger molecule (such as 6-PPDQ) would increase the distance between nAu and lower the resonance shift than smaller molecules (melamine). It is conceivable the plasmonic ruler approach to measure and detect specific compounds differing in size/molecular weight, by measuring the intensity of the resonance spectra (concentration) and the spectral shift. This would allow to determine the relative intermolecular distance between nAu as a quick and inexpensive screening test for tire wear substances.

The purpose of this research was therefore to study the resonance intensity and shifts of the plasmon resonance of nAu (2-dimensional nAu sensing) in the presence of melamine, 1,3-DPG, HMMM, and 6-PPDQ representing the main organic components in tire wear substances. This shift in plasmon resonance of citrate/Triton X-100-coated nAu was examined with the above substances alone and in mixtures to understand the interactions of these compounds at the surface the nAu. Furthermore, this methodology was used to screen for these substances in two case studies in organisms exposed to plastic pollution in urban and agriculture pollution context with freshwater mussels and biofilms.

## Methods

### Nano-Au sensor preparation and analysis

Gold nanoparticles (nAu) were purchased as citrate-coated with 10 nm diameter at 0.05 mg/mL stock solution (Nanocomposix, USA). The nAu suspension was centrifuged at 20,000 × *g* for 10 min at 4 °C to remove loosely bound or excess citrate. The pellet was resuspended in MilliQ water containing 0.001% Triton X-100. For the tire wear substances, melamine, 1,3-diphenylguanidine (1,3-DPG), hexamethoxymethylmelamine (HMMM), and 6-phenylphenyldiamine quinone (6-PPDQ) were purchased from Sigma-Aldrich (ON, Canada). Stock solutions (1 mg/mL) were prepared in methanol and subsequently diluted in MilliQ water.

For the assays, the optimized procedure for melamine with Triton X-100 was used as the starting point for analysis with the three other tire wear substances [10]. Briefly, 150 µL of nAu suspension at 50 µg/mL was mixed with 10 µL of increasing concentrations (0–30 µg/mL) of either melamine, 1,3-DPG, HMMM, or 6-PDDQ. A composite equimolar mixture of this substance at 0.1 µM was also prepared and added to nAu suspension. Following mixing for 10 s, the absorbance (resonance) spectra were taken in clear microplates between 500 and 750 nm (Neo-2 Synergy, BioTek Instrument, USA). Blanks consisted of 7% methanol in MilliQ water only and with 7% acetonitrile for the case study samples.

### Case studies

As a case study, freshwater *Elliptio complanata* mussels were caged in cylindrical nets (0.5 × 1 m) and placed for 3 months (July–October 2019) at sites in the Saint-Lawrence River downstream the city on Montréal, Québec, Canada (about 15 km from the city center), downstream (8 km) the municipal effluent plume for 90 days. Mussels were also caged at two rainfall overflow sites, which received used waters (street runoffs and untreated wastewaters) following heavy rainfall events (occurred 15–22 days during the 3-month exposure period). At the end of the exposure period, mussels were collected, placed in clean water overnight, and the digestive gland dissected out on ice. The tissues were homogenized in 100 mM NaCl, 10 mM Hepes–NaOH, pH 7.4, 1 mM DTT, and 1 µg/mL protease inhibitor (aprotinin B) using a Teflon pestle tissue grinder (5 passes at 4 °C). A 300 µL sample of the homogenate was extracted in 2.5 M NaCl and 50% acetonitrile as previously described [11]. A 10 µL sample of acetonitrile was used for the nAu-Triton X-100 as described above. The extract samples were also spiked with increasing concentration of the four tested substances following the standard addition method to compensate for matrix effects in these complex samples and determine any correspondent changes in the resonance spectral shifts.

As another case study (supplementary material), biofilms were collected during October 2023 in multiples sites gathered in the following categories: rural, agriculture, urban, and reference sites. The biofilm samples were collected on rocks using a Teflon brush and placed in 10 mL mineral water previously filtered on glass fiber filter of 0.2 µm porosity. The rural sites consisted of the north shore of Saint-Lawrence river upstream Berthierville with one border highway with no apparent upstream input of agriculture. The agriculture sites consisted of shore waters near drainage canals adjacent to cultivation fields (south shore of Lake Saint-Pierre). The urban sites consisted of downstream sites from two cities (Sorel and Saint-Ignace de Loyola of 40,000 and 3000 inhabitants, respectively). The baseline site was a river (Jacques Cartier River) located upstream the watershed from the residential area. Biofilms were extracted as described above. The data for biofilms will be in supplementary information section.

### Data analysis

The spectral analysis was repeated three times with the individual substances and mixtures of melamine, 1,3-DPG, HMMM, and 6-PPDQ. The plasmon ruler equation was used to calculate the molecular distance between nAu particles in the presence of individual compounds (melamine, 1,3-PDG, HMMM, and 6-PPDQ) and in the mixture: shift (*ʎ* − 525)/525 = 0.004 (*s*/dp)^−1.1^ where *ʎ* is the wavelength with maximum emission, *s* the distance (spacing) between nAu, and dp the diameter of particle of 10 nm in the present study [12]. For mussels, digestive glands from *N* = 8 individuals were used at each of the four sites: downstream Montreal city (DownCity), downstream the municipal effluent plume (DownEF), and two overflow sites releasing street runoffs and untreated sewage water following rainfalls. For biofilm analysis, six biofilm samples were collected at the sites specified in the previous section. The data were expressed as the mean with standard error and significance determined by rank analysis of variance followed by the Conovar-Iman test to determine significance from the reference site. Cluster analysis of plasmon resonance spectra between the intermolecular distance *s* of the four compounds and its mixtures was performed using (1 − correlation coefficient *r*) for distance. All statistical analysis was performed using the StatSoft software package. Significance was set at *p* ≤ 0.05.

## Results and discussion

**Fig. 1:**
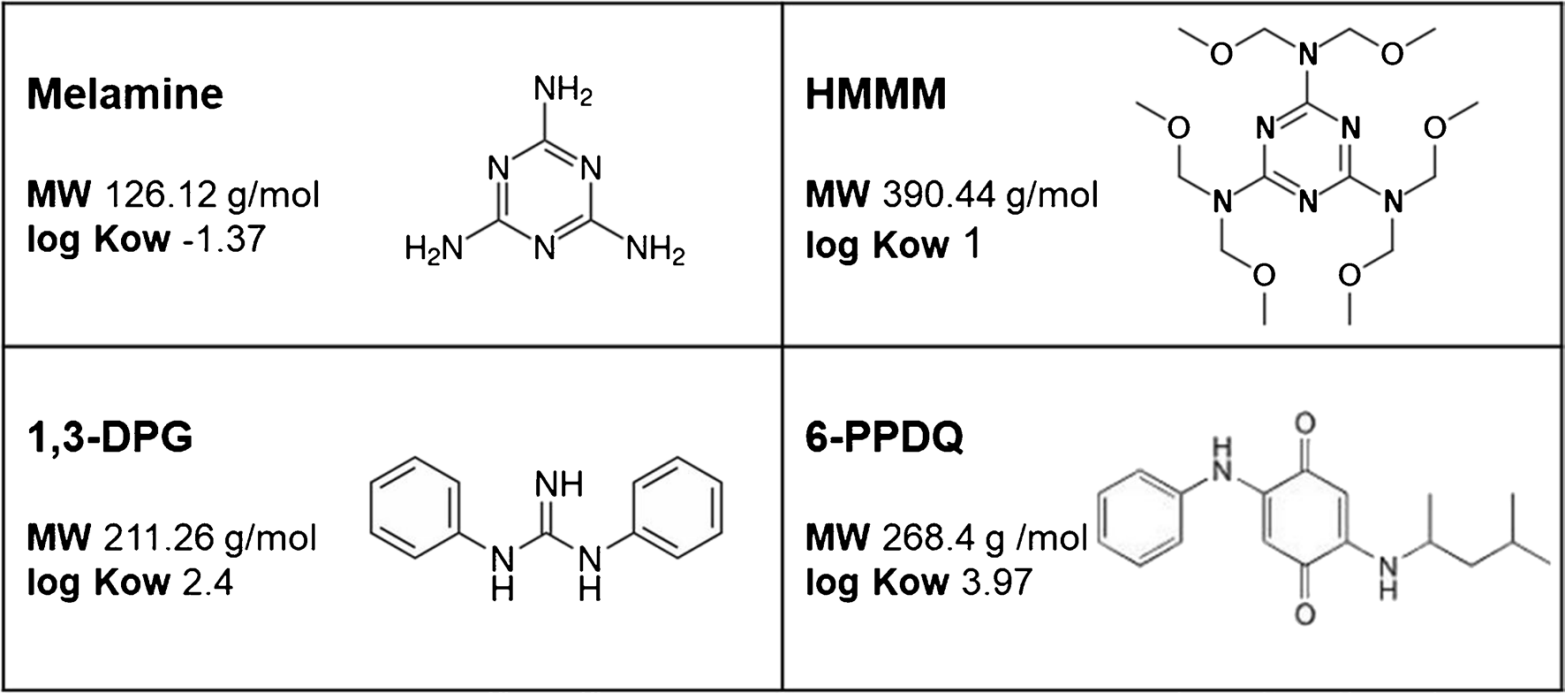
Structural properties of some tire wear substances. The chemical structure, molecular weight, and log kow (octanol–water coefficient) are shown for melamine, HMMM (hexamethoxymethylmelamine), 1,3-DPG (1–3-diphenylguanidine), and 6-PPDQ (6-phenylphenyldiamine quinone). Note the presence of melamine core in HMMM and the relative size of the compounds in increasing order: melamine < 1,3-DPG < 6-PPDQ < HMMM

To best of our knowledge, this is the first study dealing with the analysis of multiple compounds based on the plasmonic ruler approach where resonance shifts are used to discriminate the substances based on the distance between nAu. Different shifts were found between oligonucleotides and larger proteins, which provided discrimination. Plasmonic shifts were also shown to be increased with the refractive index with nAu of large aspect ratio (rod-like) permit analysis in the near-infrared spectrum [13]. However, the plasmonic ruler principle (where the spectral shift is proportional to distances between nAu) was not tested for smaller molecules. The methodology was developed using the citrate-Triton X-100-coated nAu previously developed for melamine [10]. This approach was validated using HPLC-mass spectrometry for milk products [14]. The chemical structure of the selected tire substances is reported in Fig. 1. It is noteworthy that they all contain either primary or secondary amine able to interact with the surface of nAu and form bridges between nAu facilitating aggregation and resonance shifts. The relative size of these substances based on molecular weight was as follows: HMMM > 6PPDQ > 1,3-DPG > melamine. Based on the structural geometry, melamine is also much smaller and compact in size with its aromatic heterocycle with a “longitudinal” length of 5 carbon and nitrogen atoms, compared to 6-PPDQ with a longitudinal length of 14 carbon and nitrogen atoms. The compound 1,3-DPG was larger than melamine, with a longitudinal length of 11 carbon and nitrogen atoms. This was followed by HMMM (larger than 1,3-DPG in mass) but with the same longitudinal length of 11 carbon, oxygen, and nitrogen atoms and contained an aromatic melamine core. Theoretically, the presence of these substances on the surface of nAu should increase the spacing (*s*) between nAu in the following order: melamine < 1,3-DPG ~ HMMM < 6-PPDQ.

**Fig. 2:**
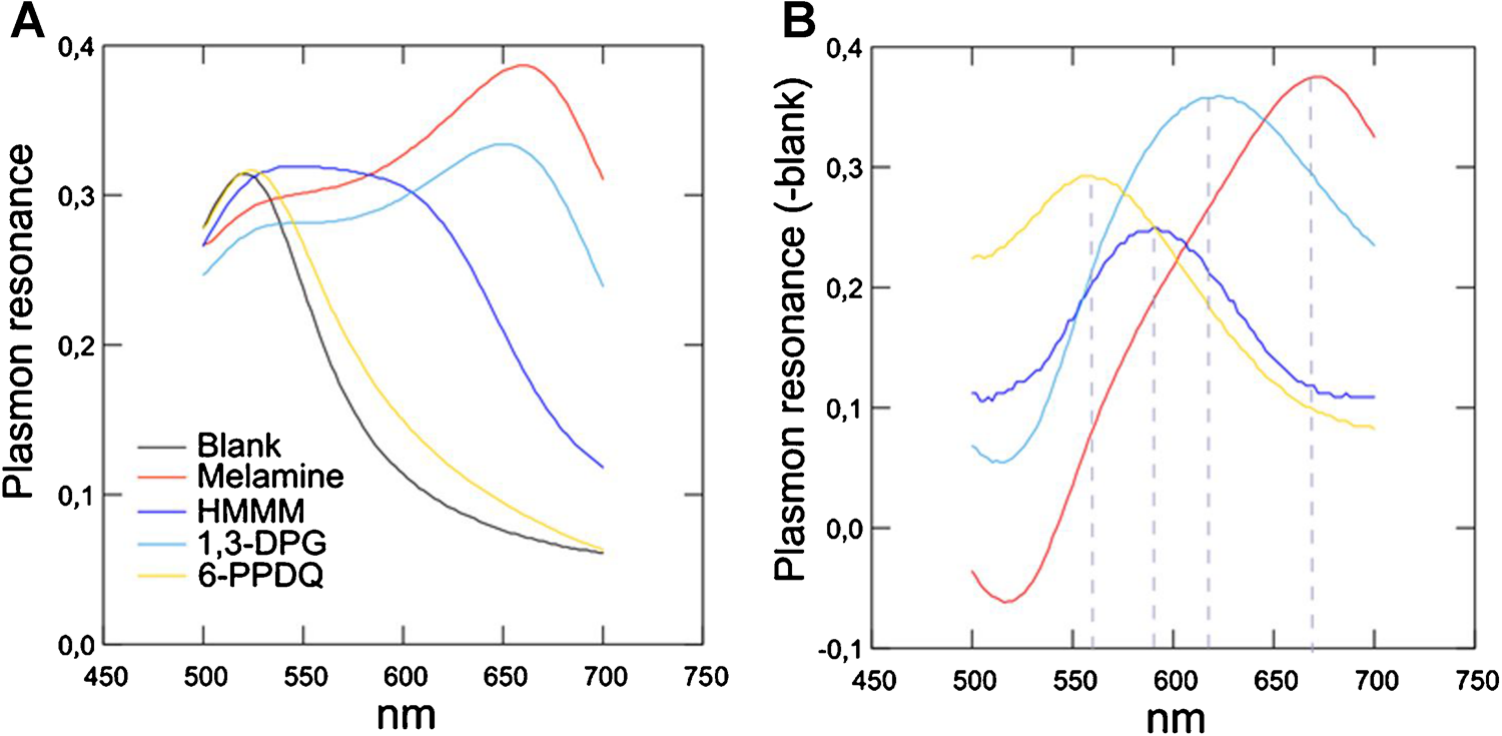
Plasmon resonance analysis of citrate/Triton X-100 nAu sensor. The plasmon resonance spectra were recorded between 500 and 700 nm in the presence of Triton X-100/citrate nAu alone and in the presence each substance (**A**). The net plasmon resonance spectra (minus blank) at 0.1 µM for each substance are shown (**B**) with peak (maximum) spectra for melamine (660 nm), 1,3-DPG (620 nm), HMMM (590 nm), and 6-PPDQ (560 nm)

The plasmonic ruler equation, which measures distance between nAu based on the resonance shift from dissociated nAu at 525 nm, indicates that the spectral shift should follow the above where the blue shift should be higher with the smallest compound melamine (nAu closer together) and so forth. This was the case with melamine, showing a maximum resonance peak at 660 nm (Fig. 2A). The plasmon resonance spectra were also determined for 1,3-DPG, HMMM, and 6-PPD quinone (Fig. 2A). Following the addition of 5–10 mM HCl, the controls (Triton X-100/citrate nAu) remain red with a resonance peak at 525 nm corresponding to non-aggregated state of nAu. The 525 nm maximum corresponds to intermolecular distance (*s*) corresponding to circa 1.5 times the diameter of nAu (10 nm in the present study) and is considered as free monomers (not aggregated) [12]. In the presence of these four substances, the plasmon resonance peak is displaced toward longer wavelengths as the distance decreases between the nAu and corresponding substance. This can be observed visually by the formation of blue color and the wavelength shift inversely related to lower size of tested substances as follows: 6-PPDQ < HMMM < 1,3-DPG < melamine (Fig. 2A). Although HMMM and 1,3-DPG share the same number of atoms, 1,3-DPG seems to bring nAu closer perhaps by forming stronger bridges between nAu. It is possible that the heavier HMMM containing tertiary amino groups is less able to form bridges by electrostatic interactions between amine electrons and the positive surface of nAu. Correction from the control revealed a maximum resonance peak at 560, 590, 620, and 660 nm for 6PPDQ, HMMM, 1,3-DPG, and melamine, respectively (Fig. 2B). By using the plasmon ruler equation, this corresponds to intermolecular distances between nAu of 0.8, 0.45, 0.3, and 0.2 nm, respectively. This suggests that these compounds could be detected when used individually during exposure to individual compounds or at site heavily contaminated with abundant substances such as HMMM. Considerable spectral overlap exists between them, which complicates the analysis of mixtures. It is noteworthy that HMMM in tire wear leachates represent one the most abundant organic compounds, with various transformation products having concentrations above 10 µg/L in rivers [6]. On the other hand, 6-PPDQ can be found at concentrations as high as 2.71 µg/L in street runoffs and 309 µg/kg in road-side soils [15]. Hence, this quick and inexpensive method has some merit as a semi-quantitative test to detect these compounds in environmental samples.

**Fig. 3:**
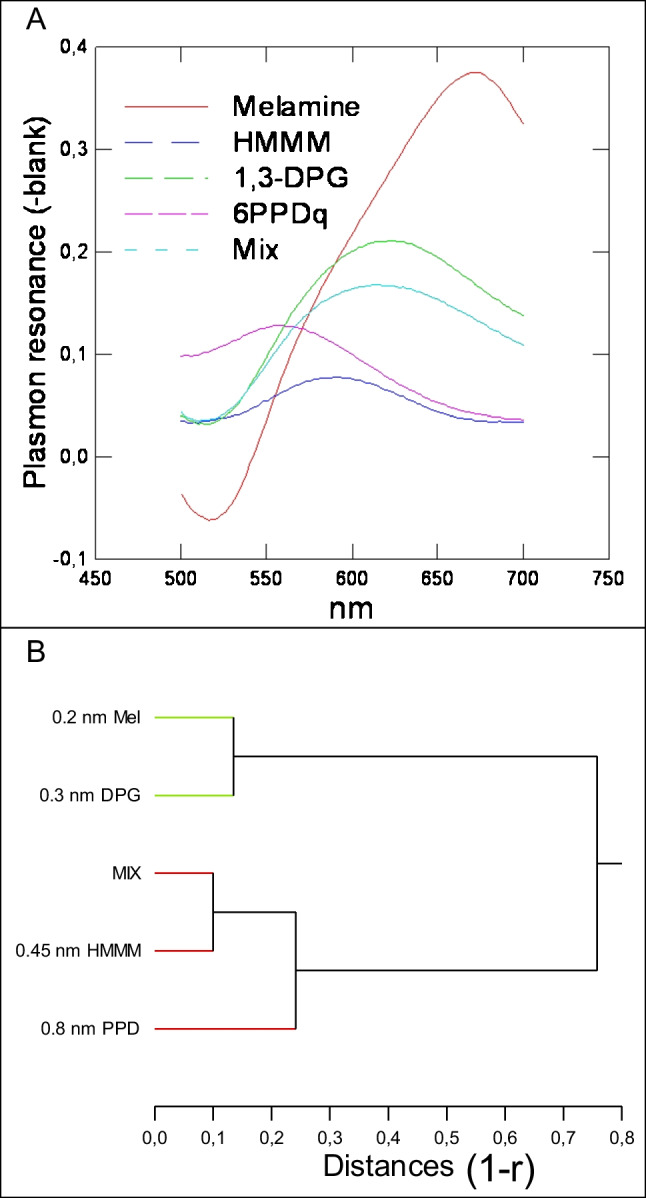
Analysis of tire wear substances mixture using the nano-Au sensor. Plasmon resonance spectra of individual compounds (melamine, 1,3-DPG, HMMM, and 6-PDDQ) alone and in mixture (**A**). Cluster analysis of emission spectra data in relationship with the plasmon ruler equation: shift (*ʎ* − 525)/525 = 0.004 (*s*/dp)^−1.1^ (**B**)

In the context that these four substances are found as mixtures in the environment and in biota, we examined the plasmon resonance spectra of an artificial mixture composed of melamine, 1,3-DPG, HMMM, and 6-PPDQ at 0.1 µM each (Fig. 3A). The mixture showed a broad peak between > 530 and 750 nm, with a maximum around 600 nm, and the peak of the individual substances was not resolved. Calculation of the intermolecular distances of the mixture at 600 nm revealed a net distance of 0.4 nm, which is close to the mean computed distance obtained with the four selected substances at 0.43 nm (Fig. 3B). This suggests that the four selected substances react randomly on the nAu particles’ surface, with a mean intermolecular distance of 0.4 nm for nAu. This result also indicates that a mixture of tire wear substances would obscure the intermolecular distances between the nAu and the identification of individual substances. The absorbance values of each tested substances individually and in mixtures are displayed in Table 1. An operational detection limit was estimated between 0.6 and 2.5 ng/mL depending on the substances, with melamine being the most sensitive. The data revealed overlapping of resonance from each substance at 560, 590, 620, and 660 nm wavelengths. The mixture showed the strongest signals at 590 and 620 nm corresponding to HMMM and 1,3-DPG. Hierarchical tree analysis revealed that the mixture was significantly correlated with the 1,3-DPG (*r* = 0.95) and HMMM (*r* = 0.6) resonance spectra, which occupies the center of the spectrum (Fig. 3B). These data complicate the semi-quantitative analysis of these products in complex mixtures/extracts and require an internal calibration approach.

**Table 1:** Plasmon resonance analysis of tire wear compounds. Each component was tested alone at a concentration of 0.1 µM. Mix 1 = mixture of all four components at 5 µg/mL each. Mix 2 = mixture of all four components at 10 µg/mL each

Compounds	A520	A560	A590	A620	A660	Detection limit^1^
Blank (nAu alone)	0.42	0.299	0.21	0.167	0.12	–
Zero	0	0	0	0	0	–
Melamine	0	0.08	0.195	0.272	**0.367**	0.7 ng/mL
1,3-DPG	0	0.12	0.19	**0.22**	0.18	1 ng/mL
HMMM	0	0.06	**0.1**	0.065	0.039	2.5 ng/mL
6-PPDQ	0	**0.128**	0.105	0.078	0.047	1 ng/mL
Mix 1 (5 µg/mL)	0	0.114	***0.159***	***0.170***	0.145	–
Mix 2 (10 µg/mL)	0	0.173	***0.235***	***0.243***	0.187	–

^1^Detection limit defined as the concentration corresponding to 2 × standard deviation of the blank

Values in bold indicate the maximal signal

**Fig. 4:**
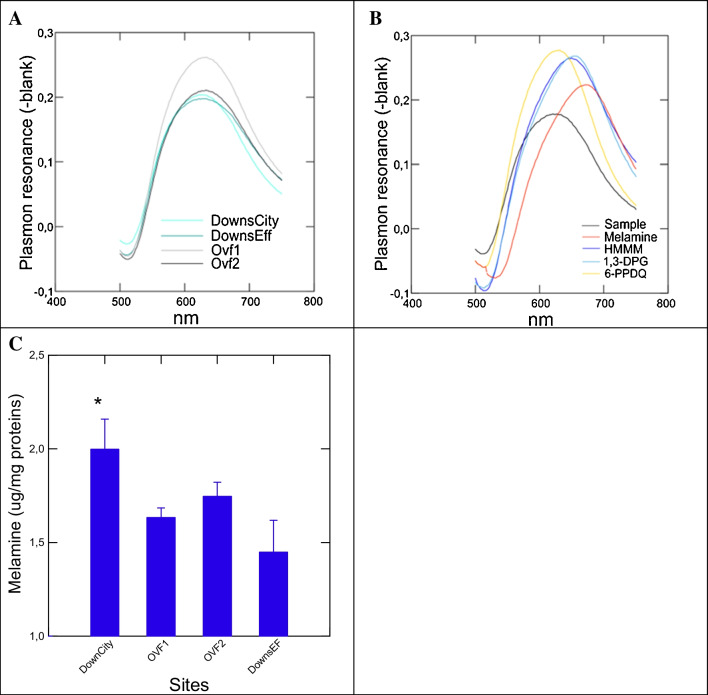
Standard addition analysis of tire wear products and HMMM levels in freshwater mussels exposed to urban pollution. Mussels were caged and exposed for 3 months during the summer of 2019 at a site downstream the city of Montréal (DownCity), downstream 8 km the effluent plume (DownEF), and two sites near rainfall overflows (OVF 1 and 2), which received only rainfalls following heavy precipitations (circa 15–22 days released raw rainwater during the 3 months exposure). The mussels were collected, placed in clean water overnight, and the digestive gland extracted using NaCl/ACN as described in the “Methods” section. The plasmon resonance spectra of digestive gland extracts (**A**), standard addition with the selected compounds at 1 µg/mL (**B**), and relative levels of HMMM in the digestive gland of mussels at the various sites based on the standard addition method (**C**). The star symbol * indicates significance from the DownEF site (treated municipal effluent plume)

To test the plasmon ruler approach, the digestive gland extracts were prepared using the NaCl/acetonitrile procedure and analyzed with the internal standard addition method (Fig. 4). The resonance spectra revealed that samples collected at the various sites showed a broad peak between > 530 and 700 nm (Fig. 4A), not so unsimilar to the artificial mixture. An internal standard addition strategy was used in an attempt to detect the presence of these four substances in mussel digestive gland extract (Fig. 4B). The addition of each of the four substances in the samples showed a gradual displacement to the corresponding resonance of tested substances (melamine, 1,3-DPG, and 6-PPDQ) and was increased in a concentration-dependent manner. This method was applied to caged mussels at various urban sites (Fig. 4C). Freshwater mussels caged at the four sites revealed that mussels located downstream a large city and the street runoffs overflow sites had elevated levels of melamine in their tissues compared to mussels from the treated municipal effluent plume. This is consistent with the high levels of melamine stemming from tire wear in streets in urban area [6]. Various transformation products of HMMM were also detected due to loss or oxidation of methoxymethyl groups and could interact at the surface of nAu. However, these interactions are beyond the resolution of the plasmon ruler methodology. The melamine assay using citrate/Triton X-100 methodology was previously confirmed by HPLC-mass spectrometry in milk products suggesting that this methodology could be used as final assay for melamine [14]. However, the other substances (HMMG, 1,3-DPG, and 6-PPD quinone were not validated by HPLC-mass spectrometry; hence, the proposed nAu assay determines only the relative level tire wear substances in complex samples. It is noteworthy that municipal effluents could reduce the levels of HMMM and other tire wear chemicals in the effluents [16], as found for suspended matter and polystyrene nanoparticles in mussels using mercaptoundecanoic acid modified nAu [14, 17].

This approach was also tested on natural biofilms collected from different anthropogenic sources of pollution (road runoffs, agriculture, and urban pollution). Biofilms collected from the reference site showed similarly a low-intensity and broad peak with maximum around 600 nm, similar to the artificial mixture of the selected four tire wear chemicals (Figure S1, supplementary file). The resonance value shifted to 590 nm with the addition of HMMM and increased with added amounts (Figure S1B suppl). Biofilms at urban or agriculture area showed an increase in the resonance spectra and revealed a significant increase in HMMM levels compared to the reference site (Figure S1C suppl). The occurrence of melamine was detected in biofilm at 0.06 µg/g in stormwater ponds with an estimated bioconcentration factor of 70 [18]. In the present study, the relative levels of HMMM in biofilms were found between 0 and 33 µg/g biofilms (5 and 95% percentile) using the nAu method, concentrations which were within the range of melamine levels from the previous study albeit at higher levels [18]. This methodology provides a simple and quick mean to detect tire wear substances in polluted environments. Detection of these compounds can be observed visually and semi-quantitative information by spectral analysis (absorbance scanning between 500 and 750 nm) using internal standards of the desired compounds.

On the one hand, HMMM appears to be one of the main tire wear contaminants with multiple degradation products, while melamine, 1,3-DPG, and 6-PPDQ are found at lower concentrations in the environment. This suggests that HMMM may serve as a tracer for tire wear pollution in biota, although less toxic than the other less polar substances. Indeed, based on the log kow, the toxicity of 6PPDQ should be higher compared to melamine and HMMM [5]. The 96-h fish toxicity of 6-PDDQ is between 0.1 and 1 µg/L in salmonids [7], while the LC50 of melamine and HMMM was estimated between 50 and 500 mg/L based on the kow. The maximum reported value of 6-PPDQ was 2.71 µg/L in runoff waters, suggesting that toxicity is likely in these contaminated urban sites. It was shown that HMMM and melamine in treated municipal effluents reached 35 and 20 µg/L, respectively [4], concentrations which should not be acutely toxic. However, the long-term/chronic effects of these compounds are presently less understood. Because melamine has a low kow, it is not expected to bioaccumulate in fish (BAF < 3) [19]. Overall, HMMM and melamine compounds represent good tracers of tire wear contamination, since they are most abundant in urban wastewaters, receiving waters downstream effluent discharges and in road runoffs. The maximum levels of 6-PPDQ reached 1.6 µg/L representing circa 22 times less the concentration than the maximum reported levels (35 µg/L) for HMMM in road runoffs [4, 20]. In respect to melamine and its derivatives (ammeline, ammelide, cyanuric acid), a study measured 223 water samples and found concentrations up to 2.7, 3.7, 1.7, and 0.2 µg/L in wastewaters, river, lake, and tap water, respectively [21]. In wastewaters and river, the maximum reported concentration reached 3.7 µg/L, which represents an important source of chronic pollution. The toxicity of melamine is considered low in aquatic organisms such as algae, fish, and shrimps, with acute effects in the mg/L range [22, 23, 24]. In respect to 1,3-DPG, the reported maximum values reached 40, 58, and 8.5 µg/L in pavement, roadsides, and farmland runoffs [19]. However, the predicted toxicity of 1,3-DPG based on the log kow indicates toxicity around 1–10 mg/L in fish [5]. Given that the bioconcentration factor could reach 30-fold [19], urban wastewaters could present a risk of chronic exposure to aquatic organisms when considering the maximum values found in wastewaters.

In conclusion, a quick (5 min reaction time) and inexpensive screening tool is presented for the evaluation of tire wear substances such as 6-PPDQ, HMMM, 1,3-DPG, and melamine. By using the plasmon ruler equation, the above compounds were shown to have specific resonance maximum, although spectral overlap was observed in a mixture at equal concentration among the compounds. In the context of analysis in complex mixtures, the relative levels could be determined using standard addition method at the prescribed resonance line. The two case studies on freshwater mussels and biofilms exposed to urban pollution revealed increased levels in melamine and HMMM, respectively. Treated wastewaters were shown to mitigate the concentration of melamine compared to other sites, such as street runoff overflow sites and downstream a large city. Moreover, this simple assay can be run on site with the aid of smartphone camera and free uploaded color analysis software.

### Supplementary Information

Below is the link to the electronic supplementary material.

Supplementary file1 (DOCX 220 KB)

## Data Availability

The data will be available upon request to the corresponding author.
