# Supplementary material for: A nanogold sensor test for tire wear chemicals based on the plasmon ruler approach
Source: Mikrochim Acta. 2024 May 17;191(6):335. doi: 10.1007/s00604-024-06376-3 (PMC11101529; doi:10.1007/s00604-024-06376-3)
Supplement: Supplementary file 1 — Supplementary file1 (DOCX 220 KB) [file 604_2024_6376_MOESM1_ESM.docx]

| 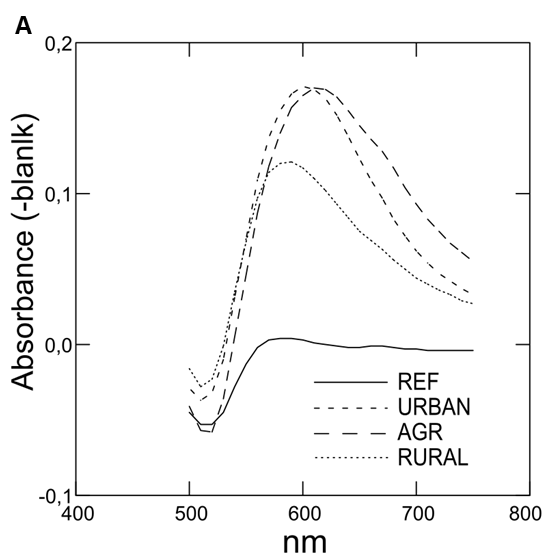 |  |
| --- | --- |
| 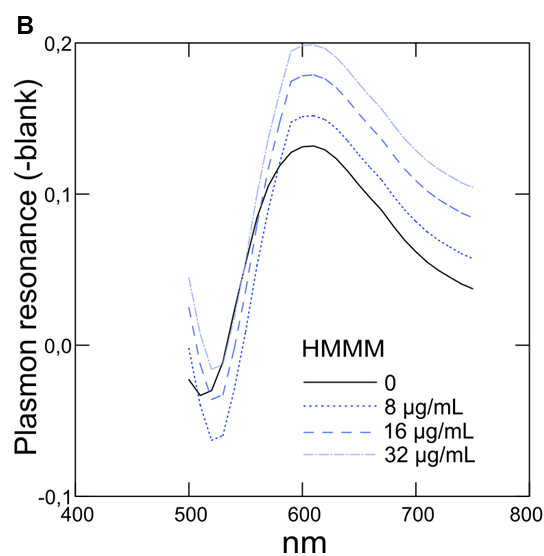 | C   |

**Figure 1S**. Standard addition of HMMM in biofilm acetonitrile extract.

Biofilms were extracted in 2.5 M NaCl and acetonitrile and analyzed using the plasmonic nAu sensor. The spectrum of various biofilm extracts from different area (reference, rural, agriculture and urban) is shown (**A**). A biofilm extract pool from agriculture and urban areas were analyzed using the standard addition method with HMMM (**B**) and reported HMMM levels in biofilms (**C**). The data represent the mean with the standard deviation. The star symbol * indicates significance from the reference site.
